# The Farsi version of the Hypomania Check-List 32 (HCL-32): Applicability and indication of a four-factorial solution

**DOI:** 10.1186/1471-244X-11-14

**Published:** 2011-01-20

**Authors:** Mohammad Haghighi, Hafez Bajoghli, Jules Angst, Edith Holsboer-Trachsler, Serge Brand

**Affiliations:** 1 Research Center for Behavioural Disorders and Substance Abuse of Hamadan University of medical sciences, Hamadan, Islamic Republic of Iran; 2 Iran University of Medical Sciences, Tehran, Islamic Republic of Iran; 3 Zurich University Psychiatric Hospital, Zurich, Switzerland; 4 Psychiatric Hospital of the University of Basel, Basel, Switzerland

## Abstract

**Background:**

Data from the Iranian population for hypomania core symptom clusters are lacking. The aim of the present study was therefore to apply the Farsi version of the Hypomania-Check-List 32 (HCL-32), and to explore its factorial structure.

**Methods:**

A total of 163 Iranian out-patients took part in the study; 61 suffered from Major Depressive Disorder (MDD), and 102 suffered from Bipolar Disorders (BP). Participants completed the Mood Disorder Questionnaire (MDQ) and the Hypomania Checklist (HCL-32). Exploratory factor analyses were used to examine the properties of the HCL-32. A ROC-curve analysis was performed to calculate sensitivity and specificity.

**Results:**

The HCL-32 differentiated between patients with MDD and with BP. Psychometric properties were satisfactory: sensitivity: 73%; specificity: 91%. MDQ and HCL-32 did correlate highly. No differences were found between patients suffering from BP I and BP II.

**Discussion:**

Instead of the two-factorial structure of the HCL-32 reported previously, the present pattern of factorial results suggest a distinction between four factors: two broadly positive dimensions of hypomania ("physically and mentally active"; "positive social interactions") and two rather negative dimensions ("risky behavior and substance use"; "difficulties in social interaction and impatience").

**Conclusion:**

The Farsi version of the HCL-32 proved to be applicable, and therefore easy to introduce within a clinical context. The pattern of results suggests a four factorial solution.

## Background

There is evidence that bipolar disorders have been under-diagnosed (cf. [1]), and recent findings suggest that bipolar disorders are increasing among children and adolescents [2]. However, increased efforts are being made to overcome the lack of research and instruments [3,4]. In this respect, the Hypomania Check-List 32 (HCL-32; [5]) has gained considerable importance. For instance, the HCL-32 has been applied with adolescents [6-8], with a non-clinical sample of young adults [9], and with a broad range of patients suffering from affective disorders in Europe, South America, and the Far East [1,3]. In this respect, Carta et al [10] were able to show in a clinical sample that the HCL-32 was a sensitive screening instrument for bipolar disorder in a psychiatric setting. Currently, a short version consisting of 16 instead of 32 items is being validated [4], and recently, the HCL-32 has been used to screen patients suffering mood disorders more generally [11]. However, for the Persian (or Farsi) language area, research is scare and this holds particularly for the Islamic Republic of Iran. In Iran, it is estimated that at least 7 million people (9.43% of the population) suffer from one or more psychiatric disorders [12], while the mental health pattern in Iran is similar to that of western countries [12]. Bipolar disorders, however, are under-investigated in this country. To address this lack of research, the aim of the present study was four-fold: 1) to introduce a Farsi version of the Hypomania-Check-List-32 (HCL-32; [5]), a self-rating questionnaire to assess hypomania; 2) to determine whether the HCL-32 allows a distinction between patients with Major Depressive Disorder (MDD) and Bipolar Disorder (BP), and between patients with BP I (periods of depressive and manic stages) and BP II (periods of depressive and hypomanic stages) disorders; 3) to compare the data with those from an established questionnaire (Mood Disorder Questionnaire: MDQ; [13,14]), and 4) to explore the factorial properties of the Farsi version.

## Method

The study was conducted at the Iran University of Medical Sciences, Tehran, and the Research Center for Behavioural Disorders and Substance Abuse of Hamadan University of Medical Sciences, Hamadan. The study was approved by the Hamadan ethical committee (Iran). Written informed consent was obtained from each participant before inclusion.

### Patients

A total of 179 out-patients were approached. Patients were included if they were willing and able to participate and to complete the questionnaires, and if experts' ratings diagnosed MDD or BP according to the DSM-IV. Of the patients approached, nine (5%) were excluded due to comorbid disorders (substance abuse). 170 agreed to participate at the first interview (95%), and 163 (91%) completed the questionnaires correctly. Of these, 61 suffered from Major Depressive Disorder (MDD) and 102 suffered from Bipolar Disorder (BP I; n = 59 and BP II; n = 43). Clinical characteristics of the patients are shown in Table 1.

**Table 1:** Clinical characteristics of the sample

		Samples		Statistics
	MDD	BP I	BP II	

*N*	61	59	43	

Male/female	25/36	35/24	24/19	*X*^*2*^(2) = 4.17, *p *= .11

Mean age (SD)	35.60 (12.35)	35.12 (10.35)	36.00 (15.21)	*F*(2, 160) = 0.06, *p *= .94

Clinical state during interview:				

Recovery	34/61 (56%)	34/59 (58%)	21/43 (49%)	*X*^*2*^(2) = 0.83, *p *= .66

MDD	26/61 (43%)	23/59 (39%)	18/43 (42%)	*X*^*2*^(2) = 1.07, *p *= .59

Mania	0/61 (0%)	5/59 (8%)	0/43 (0%)	*X*^*2*^(2) = 9.10, *p *= .01

Hypomania	1/61 (2%)	3/59 (5%)	5/43 (12%)	*X*^*2*^(2) = 1.46, *p *= .48

Age at onset of illness (years: M (SD)	32.63 (10.92)	29.74 (8.89)	31.00 (11.09)	*F*(2, 160) = 1.19, *p *= .31

Duration of illness (years: M (SD)	3.78 (3.99)	5.34 (4.23)	6.39 (6.14)	*F*(2, 160) = 4.04, *p *= .02

Number of affective episodes	1.74 (0.87)	2.36 (1.24)	3.65 (1.60)	*F*(2, 160) = 30.76, *p *= .00

MDD = Major Depressive Disorder; BP I = bipolar disorder I; PB II = bipolar disorder II; M = mean; SD = standard deviation

As shown in Table 1, the three groups did not differ with respect to gender distribution, age or age at onset of illness, but did differ with respect to the duration of illness and the number of affective episodes.

### Instruments

Experts at the two study centres diagnosed patients based on DSM-IV criteria [15]. To do so, a psychiatric interview was conducted using the SCID (Structured Clinical Interview for DSM Disorders [16] and the Schedule for Affective Disorders and Schizophrenia (SADS; [17]). Afterwards, patients completed the Mood Disorders Questionnaire (MDQ;[13], Farsi version: [14,18]). The MDQ assesses bipolar disorders and consists of 13 items focusing on the occurrence of mood changes (answers: yes (= 1) or no (= 0)), the occurrence of mood disorders within the same period of time, and the possible adverse impact of mood changes on everyday life. Psychometric properties of the Farsi version have been shown to be robust and satisfactory [14,18]. Higher scores reflect increased occurrence of bipolar disorders. Cronbach's alphas: entire sample:.85; patients with MDD:.82; patients with BP I and II:.88.

Next, patients also completed the Hypomania-Check-List 32 [5]. The HCL-32 consists of 32 statements concerning behavior (e.g., "I spend more money/too much money"), mood (e.g., "My mood is significantly better"), and thoughts (e.g., "I think faster") within the last four weeks. Answers are "yes" (= 1) or "no" (= 0), and higher scores reflect more marked hypomanic states. Cronbach's alphas: entire sample:.84; patients with MDD:.82; patients with BP I and II:.90. Cronbach's alphas thus do imply a high degree of internal consistency. To ensure optimal translations, we rigorously followed the procedure proposed by Brislin ([19]; cf. [1]); that is to say, the English items were translated into Farsi, and then back-translated into English by an independent translator. Consensus was reached on a final version that was subjected to the translation-retranslation process.

Overall, patients needed about 15 minutes to complete the two questionnaires.

### Statistical analyses

Pearson's correlations were computed to compare the sum scores between MDQ and HCL-32. To test for differences between patients with MDD and BP with respect to the MDQ and HCL-32, instead of the classical Student's t-test the more robust Welch-test ''w'' was used [20,21]. Single Welch-tests were also used to compare the present data with results from historical samples as reported in Angst et al. [1]. The HCL-32 items were submitted to factor analysis with orthogonal rotation. Logistic regression and ROC curve analysis were performed to estimate the sensitivity and specificity of HCL-32 as a screening method to discriminate between patients with MDD and those with BP.

Test results with an alpha level below .05 were reported as significant. However, we placed more emphasis on effect sizes (*d*) following Cohen's advice [22,23] that the importance of *p*-values should not be overestimated. Effect sizes for *t*- and *w*-tests were calculated following Cohen [22], with 0.49 ≥ *d *≥ 0.20 indicating small (i.e., negligible practical importance), 0.79 ≥ *d *≥ 0.50 indicating medium (i.e., moderate practical importance), and *d *≥ 0.80 indicating large (i.e., crucial practical importance) effect sizes.

## Results

### General results

The relation between HCL-32 and MDQ scores was statistically significant (entire sample: *r *= .68, *p *< .01; patients with MDD: *r *= .61, *p *< .01; patients with BP: *r *= .72, *p *< .001).

Compared to patients with MDD, patients with BP had both higher HCL-32 scores (MDD: *M *= 16.26, *SD *= 9.39; BP: *M *= 19.83, *SD *= 5.50: *w*(111.97) = 2.62, *p *= .01, *d *= 0.59), and higher MDQ scores (MDD: *M *= 7.77, *SD *= 3.29; BP: *M *= 9.80, *SD *= 3.95: *w*(144.23) = 1.79, *p *= .04, *d *= 0.51). No differences were found for HCL-32 and MDQ scores between patients with BP I or BP II (*w*s < 0.88, *p*s > .38).

### Comparison of the HCL-32 scores of the Iranian sample with data from samples of patients suffering from MDD and BP from Northern Europe, South America and East Asia

Statistical characteristics of Northern European, South American and East Asian were taken from Angst et al. (2010) [1]. Compared to samples from Northern Europe, South America and East Asia, the Iranian patients with MDD did not differ in HCL-32 scores. Compared to samples from Northern Europe, South America and East Asia, the patients with BP did have higher scores, though effect sizes were small to medium, indicating negligible to medium practical importance (see Table 2).

**Table 2:** Statistical comparison of the HCL-32 data between Iranian out-patients and patients suffering from major depressive disorders (MDD) and bipolar disorder (PB) from other countries.

	Samples from other countries		
	**Northern Europe**	**South America**	**East Asia**

*N*	672	423	631
HCL-32 total score (*M *and *SD*)	17.10 (6.00)	16.45 (6.05)	15.50 (6.70)

			
Iranian sample			
MDD (*N *= 61)			
HCL-32 total score (*M *and *SD*)	17.26 (6.39)	17.26 (6.39)	17.26 (6.39)
t-tests (*df *= 60)	*t *= 0.20; *p *= .84	*t *= 0.99; *p *= .32	*t *= 2.15; *p *= .04^1^
Effect sizes *d*	0.025	0.085	0.268^1^
			
BP (*N *= 102)			
HCL-32 total score (*M *and *SD*)	19.83 (5.50)	19.83 (5.50)	19.83 (5.50)
t-tests (*df *= 101)	t = 8.96; p = .000	*t *= 6.21; *p *= .000	*t *= 7.95; *p *= .000
Effect sizes *d*	0.47	0.58	0.71

Notes: HCL-32 = Hypomania Check-List 32. MDD = major depressive disorders; BP = bipolar disorders. ^1 ^Note that even if the p-value suggests a significant mean difference, the effect size of 0.268 indicates that the mean difference was small and of negligible practical importance.

### Sensitivity and specificity of the HCL-32 scores with respect to the diagnoses

After binary logistic regression with MDD and BP as a dependent variable and HCL-32 scores as an independent variable, sensitivity, i.e., the number of subjects correctly identified with MDD, was found to be 73%, whereas specificity, i.e., the number of subjects correctly identified with BP, was found to be 91%, corresponding to an overall precision of 82%. The optimal cut-off point was 14.5. Applying this cut-off, 81% of the patients with BP were above the cut-off score (patients with MDD: 31% were above the score. For a cut-off score of 7 for the MDQ: patients with BP: 79%; patients with MDD: 28%. Considering the AUC (area under the curve) value of 0.81 of the ROC curve, this result was at the middle, but still satisfactory, limit for heuristic approaches (cf. [24]).

### Reducing the 32 items to factors

The first ten factors extracted by the factor analysis had eigenvalues greater than 1, together accounting for 68% of the overall variance. However, following Brown [25], a further item selection was performed as follows: items were excluded if they loaded on more than one factor (i.e., cross-loadings), or if they showed small loadings on all factors (i.e., low communalities). On this basis ten out of 32 items were excluded. A factor analysis of the 22 remaining items yielded four factors with eigenvalues greater than 1, together accounting for 78% of the variance. The first factor, labelled "Positively physically and mentally active" had an eigenvalue of 4.29; for the second factor, labelled "Positive social interactions", the eigenvalue was 3.49; for third factor, labelled "Risky behavior and substance use", the eigenvalue was 2.35; for the fourth factor, labelled "Difficulties in social interaction and impatience" the eigenvalue was 1.56 (see Table 3). The first two factors may be considered positive dimensions ("bright" or "sunny" side of hypomania), the latter two factors may be considered negative dimensions ("dark" side of hypomania).

**Table 3:** Items of the HCL-32 and their allocation to four factors.

	Factors			
	**Favorable dimensions**		**Unfavorable dimensions**	

	**Physically and mentally active**	**Positive social interactions**	**Risky behavior and substance use**	**Difficulties in social interaction and impatience**

I am physically more active	**.675**	.189	-.059	.094
I engage in lots of new things	**.636**	.080	.180	-.083
I enjoy my work more	**.623**	.122	-.053	-.161
I am more interested in sex/..have increased sexual desire	**.608**	-.083	.137	.367
I am more confident	**.605**	.374	-.065	.001
I have more ideas	**.526**	.221	.322	-.067
I think faster	**.593**	.114	.063	.272
I do things more quickly	**.500**	.360	.025	-.208
I feel more energetic	**498**	.278.	.111	.003

I talk more	.155	**.656**	.089	.193
I am more sociable	.211	**.618**	.032	-.233
I am less shy	.003	**.563**	.320	.054
I want to meet or do actually meet more people	.180	**.559**	-.037	.093

I tend to drive faster	.065	.061	**.661**	-.006
I drink more coffee	.025	.085	**.617**	-.135
I drink more alcohol	.032	.107	**.581**	.137
I take more risks in my daily life	.182	-.171	**.560**	.462
I smoke more cigarettes	.009	.059	**.499**	.133

I can be exhausting or irritating for others	.164	-.035	.059	**.688**
I get into more quarrels	-.062	.158	.129	**.627**
I am more impatient/...get irritable more easily	-.270	.028	-.070	**.539**
My thoughts jump from topic to topic	-.078	.265	.072	**.462**

Note: Bold factor loadings refer to the corresponding factors.

## Discussion

The main results of the present study are that the Farsi version of the HCL-32 did correlate highly with an existing self-rating questionnaire for bipolar disorders (MDQ), that it discriminated between patients with MDD and BP, that mean scores did not substantially differ from those of samples drawn from other continents, and that contrary to previous findings, a four-factorial, rather than a two-factorial solution emerged.

Strong correlations between the established Farsi version of the MDQ and the present HCL-32 do suggest that the Farsi version of the HCL-32 measures the same psychological construct, hypomanic stages within bipolar disorders. Moreover, Cronbach's alphas reflected a consistently high internal consistency. Therefore, the Farsi version seems applicable for these disorders. Moreover, one needs only few minutes to complete the HCL-32; this implies that the present version is a quick and easy self-assessment tool. In this regard, the present data do also fit well within the broad range of findings which suggest a cross-cultural and generalized presence of bipolar disorders [1,3].

Whereas the present questionnaire enables discrimination of patients with MDD and patients with BP, it does not allow a distinction between patients with BP I and BP II. The underlying reasons remain unclear, though one might speculate that in the current sample differences between patients with BP I and BP II were not present at the time of the survey. Another reason may be that the mood states, rather than being categorical entities, may be better viewed within a continuum ranging from one pole (depressive symptoms) to another (manic stage; cf. [7,26]), and that within this continuum BP I and BP II stages are barely detectable by self-rating. In this view, it is also of note that previous research with the HCL-32 has not consistently allowed a distinction between BP I and BP II [1,5,27] (but see also [3]).

In contrast to previous studies (cf. [28,1,11,6,7]), a four-factor rather than a two-factor structure emerged. However, Holtmann et al. [8], applying the HCL-32 with a sample of adolescents (mean age: 17.1 years), found a three-factor structure, with the first factor ''active-elated'' reflecting symptoms related to energy and activity. By contrast, the adult factor ''irritable-risk taking'' was better reflected by two separate factors (''disinhibited/stimulation-seeking'' and ''irritable-erratic''). Importantly, these factors were associated with externalizing problems. Also differing from earlier two-factorial solutions, Rybakowski et al. [29] reported a three-factor solution for a sample of patients suffering from treatment-resistant depression. Factor 1 was related to elevated mood and increased activity, factor 2 was related to increased sexual activity, whereas factor 3 was related to irritability. In brief, it seems that the factorial structure of the HCL-32 is not conclusively limited to two factors, and that solutions may vary as a function of the sample concerned.

### Limitations

Despite the new findings, several issues warrant against generalization, and these data should be interpreted cautiously. First, the sample size is rather small and issues related to gender were not taken into account. However, we emphasized effect size calculations which are not sensitive to sample sizes. Second, comorbid substance use or dependence is relatively common in bipolar disorder, and to some degree also in depression. However, respondents with comorbid substance use were excluded from the sample. As a result, data may be biased and not entirely representative. Third, recall of hypomanic symptoms in the past as assessed by the HCL-32 and MDQ might have been biased by current clinical state. Fourth, results from comparisons with samples taken from Angst et al. [1] should be interpreted cautiously because of the uneven distribution of patients suffering from MDD and BP. Fifth, only patients willing and able to participate and to complete the questionnaires were included in the study; therefore, again, results may be biased. Sixth, the cross-sectional design does not allow investigation of further implications related to the long-term development of the assessed mood changes. Seventh, compared to other findings (e.g., [10]) the cut-off of 14.5 points to distinguish between patients suffering from MDD and BP might be rather high, though this cut-off point is comparable to other studies (cf. [5-7,9]). Last, statistical comparisons between the present data and statistical information from other samples were not systematically controlled for gender and age.

## Conclusion

The Farsi version of the HCL-32 is easy to complete and provides detailed information (on four dimensions) about what a patient thinks about her/his hypomanic stages. Therefore, the questionnaire is easily applicable within the clinical context. Future research might focus on the issue of the extent to which these four dimensions predict long-term development of patients' mood changes. Moreover, the Farsi version of the HCL-32 is also widely applicable, since about 150 million of people throughout the world use Farsi as first or second language.

## Competing interests

The authors declare that they have no competing interests.

## Authors' contributions

MH and HB translated the English version of the HCL-32 into Farsi, conducted the study, ran the experts' ratings, collected the questionnaires and supervised the study. JA provided the questionnaires and the scientific background. EHT provided the scientific background and co-wrote the manuscript. SB proposed and initiated the study, performed the statistical analyses, and co-wrote the manuscript. All authors read and approved the final manuscript.

## Pre-publication history

The pre-publication history for this paper can be accessed here:

http://www.biomedcentral.com/1471-244X/11/14/prepub
